# The effectiveness and safety of parathyroid hormone in fracture healing: A meta-analysis

**DOI:** 10.6061/clinics/2019/e800

**Published:** 2019-04-16

**Authors:** Hao Hong, Ting Song, Yang Liu, Jun Li, Qilong Jiang, Qizhi Song, Zhongliang Deng

**Affiliations:** I Department of Orthopaedics, Second Affiliated Hospital of Chongqing Medical University, Chongqing, China; II Institute of Forensic Science, Chongqing Public Security Bureau, Chongqing, China; III Department of Orthopaedics, General Hospital of Chongqing Steel Company, Chongqing, China

**Keywords:** Parathyroid Hormone, PTH, Teriparatide, Fracture Healing

## Abstract

The very large economic and social burdens of fracture-related complications make rapid fracture healing a major public health goal. The role of parathyroid hormone (PTH) in treating osteoporosis is generally accepted, but the effect of PTH on fracture healing is controversial. This meta-analysis was designed to investigate the efficacy and safety of PTH in fracture healing. The EMBASE, PubMed, and Cochrane Library databases were systematically searched from the inception dates to April 26, 2018. The primary randomized clinical trials comparing PTH treatment for fracture healing with placebo or no treatment were identified. We did not gain additional information by contacting the authors of the primary studies. Two reviewers independently extracted the data and evaluated study quality. This meta-analysis was executed to determine the odds ratio, mean difference, standardized mean difference, and 95% confidence intervals with random-effects models. In total, 8 randomized trials including 524 patients met the inclusion criteria. There were significant differences in fracture healing time, pain relief and function improvement. There were no significant differences in the fracture healing rate or adverse events, including light-headedness, hypercalcemia, nausea, sweating and headache, except for slight bruising at the injection site. We determined that the effectiveness and safety of PTH in fracture healing is reasonably well established and credible.

## INTRODUCTION

It is estimated that there are 16 million cases of fractures in the United States every year. Despite most bone having excellent regenerative ability during fracture healing, approximately 5% to 10% of fractures have complications, such as delayed healing or nonunion 1-3. Delayed healing and nonunion are defined as fractures that have not healed for 6 and 9 months, respectively 4,5. The complications result in extended treatment time, reduced quality of living and potential additional remedial surgery, which give the patients a heavy burden and have serious societal implications 1,6. Fracture repair is an intricate process regulated by numerous genes and is affected by chemokines, cytokines, growth factors and other molecules 7-9. To promote bone regeneration and prevent complications, an in-depth understanding of the fracture healing process and the adoption of suitable interventions are essential.

Parathyroid hormone (PTH) plays a vital role in bone regeneration by stimulating the differentiation and proliferation of osteoblasts and osteoclasts 10,11. Osteoporosis has been treated with intact PTH (1-84) and teriparatide, which is an N-terminal fragment (1-34) of PTH 12. Currently, teriparatide is the only anabolic bone therapeutic medicine to treat osteoporosis approved by the Food and Drug Administration (FDA) 13-16. In patients with high fracture risk, PTH can improve bone quality and prevent fractures 17.

As described previously, PTH treatment could increase bone formation biomarkers higher than it increases bone resorption biomarkers 18,19. Some animal studies have demonstrated that daily application of PTH (1-34) could increase bone mineral density to accelerate fracture healing and persistently affect the remodeling callus during fracture healing 20,21. Furthermore, studies have shown that PTH could improve fracture healing at different skeletal sites of patients 22-24. However, we found that recent studies in the literature were inconsistent regarding the effects of PTH treatment on fracture healing 25,26.

Therefore, a meta-analysis on randomized controlled trials (RCTs) was performed to demonstrate whether PTH can promote fracture healing by comparing PTH treatment to a placebo treatment or no treatment in patients with fracture.

## METHODS

This meta-analysis was conducted based on the Cochrane Handbook for Systematic Reviews of Interventions 27 and presented in compliance with Preferred Reporting Items for Systematic Reviews and Meta-analyses guidelines (PRISMA) 28.

### Search strategy

The EMBASE, PubMed, and Cochrane Library databases were systematically searched from the inception dates to April 26, 2018. The keywords and Medical Subject Headings (MeSH) were “Parathyroid Hormone”, “Hormone, Parathyroid”, “Parathormone”, “Parathyrin”, “Parathyroid Hormone Peptide (1-34)”, “PTH (1-34)”, “Parathyroid Hormone (1-34)”, “Teriparatide”, “hPTH (1-34)”, “Human Parathyroid Hormone (1-34)”, “Parathar”, “Teriparatide Acetate”, “Forteo”, “Parathyroid Hormone (1-84)” and “PTH (1-84)” in combination with “Fracture Healing”, “Healing, Fracture”, “Healings, Fracture” and “Fracture Healings”. We did not restrict the language. The detailed search strategy is reported in the supplement. We identified original RCTs and did not obtain additional information by contacting authors of the primary studies.

### Inclusion criteria

Studies were included if they met the following criteria: (A) participants, aged 21 to 94 years old, were patients with fractures and treated by PTH; (B) RCTs comparing the PTH intervention with a placebo or no treatment; (C) teriparatide was subcutaneously injected at 20 μg per day, or PTH (1-84) was administered at 100 μg per day; and (D) trials provided the relevant data.

Studies were excluded if they met the following criteria: (A) the patients had been using PTH, unless they had experienced a wash-out period; (B) there were contraindications to related drugs at any time; (C) any other antiosteoporotic drug had been taken in either the experimental group or comparison group; (D) liver enzymes were more than twice the upper limit or serum calcium was higher than the reference level; (E) patients had rheumatoid arthritis, pathologic fractures, history of tumor or chemotherapy, metabolic bone disease, chronic renal failure, or any disease affecting bone metabolism; (F) the studies were published as abstracts, reviews or letters; or (G) the articles were not available or the data had already been published.

### Risk-of-bias assessments

According to the Cochrane risk-of-bias criteria 27, 2 researchers (HH, YL) independently evaluated the methodological quality of the selected RCTs. Every quality item was classified into unclear risk, low risk, or high risk. Any disagreements about a trial were resolved through discussion or consultation with an expert. Seven items were used to assess bias, as shown in Figure 2. Other bias was defined as trials with dissimilar baseline characteristics between groups or with sponsorship coming from drug companies.

### Data extraction

The following information from each study was extracted independently by two researchers (HH, TS): authors, publication year, participant characteristics, number of cases, type of fracture, duration of trials, explicit treatment, drug dose, fracture healing-related data and adverse events. Any disagreements were resolved through discussion. We only extracted the relevant information and data from the original articles when more than 2 groups were compared in the study.

The primary endpoints were the fracture healing rate and time. When three of four fracture cortices were connected by the bone bridge shown on either lateral or anteroposterior radiographs, the fracture was considered healed 29-35. A visual analog scale (VAS) 31,34,36,37 and the Patient-Rated Wrist Evaluation (PRWE) were common methods for estimating degree of pain 33. Kinematic mobility could quantify functional outcomes using the PRWE 33, “Disabilities of the Arm, Shoulder, and Hand” scoring (DASH) 37, Johanson Hip Rating Questionnaire (JHRQ) 30, or Timed “Up and Go” test (TUG) 31. Because adverse events were reported by inconsistent methods 29,32,33,36,37, we could not pool the relevant data and only describe them in Table 7.

### Statistical analysis

Our meta-analysis was conducted in Revman version 5.3 from the Cochrane Collaboration. We calculated the odds ratio (OR) and 95% confidence intervals (CIs) using the Mantel-Haenszel statistical method for dichotomous outcomes, while we determined mean difference (MD) or standard mean difference (SMD) and 95% CIs using the inverse variance statistical method for continuous outcomes. The analyses were 2-tailed, and the data pooled with a random-effects model. *p*<0.05 was considered statistically significant. Statistical heterogeneity in the summary data was assessed with I^2^ statistics and *p*-values (I^2^>50% or *p*-value<0.10 was considered to indicate significant heterogeneity) 38,39. Sensitivity analyses were fulfilled by excluding the trials one by one. We performed subgroup analyses to appraise whether clinical characteristics could alter the results, to evaluate the statistical significance between the subgroups, and to estimate the publication bias with funnel plots.

### Grading of evidence quality

Regarding risk of bias, inconsistency, indirectness, imprecision, and publication bias, the quality of evidence was independently assessed by two authors (HH, YL) based on the Grading of Recommendations Assessment, Development, and Evaluation (GRADE) 40 methodology. The estimated results were classified as very low, low, moderate, or high. We constructed a summary table using the GRADE Profiler (version 3.6).

## RESULTS

### Literature search and characteristics

**Figure 1:**
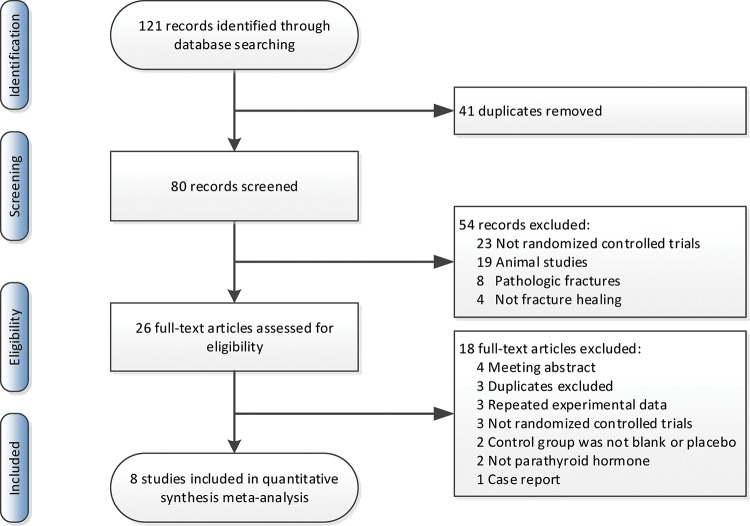
Flow diagram of the study selection process.

From the recently published studies, 121 potentially eligible RCTs were screened by titles and abstracts. There were 26 records read in full. Eventually, 8 records were in conformity with the inclusion criteria. A manual search of the reference lists within these studies did not reveal additional eligible studies. The process of screening the studies is illustrated in Figure 1.

Ultimately, in the meta-analysis, there were 524 participants from 8 RCTs, of which 79.6% were women and 20.4% were men. The mean age was 73.0 years old. For the fracture type, two trials had upper limb fractures (including distal radial fractures and proximal humeral fractures), five trials had lower limb fractures (including lower-extremity stress fracture, femoral neck fracture, trochanteric fractured neck of femur, osteoporotic intertrochanteric fractures, and hip fractures), and one trial had pelvic fracture. The general characteristics of the included studies are presented in Table 1.

The experimental groups received once-daily subcutaneous treatment with 20 μg of teriparatide or 100 μg of PTH (1-84). The anabolic effect of 20 μg teriparatide is equivalent to 100 μg PTH (1-84) because of the differences in pharmacokinetics (41). The control group received a placebo, no treatment, or other drugs. The duration of PTH treatment ranged from 4 weeks to 24 months. The study by Aspenberg et al. (33) included two experimental groups, taking 20 μg or 40 μg teriparatide. In the study by Huang et al. (34), the two experimental groups could take either 20 μg teriparatide or 20 μg teriparatide plus 70 mg alendronate. Additionally, the study by Kanakaris et al. (30) had two control groups that took either vitamin D and calcium or vitamin D and calcium plus 70 mg alendronate. We excluded those ineligible groups. The detailed characteristics are shown in Table 2.

### Risk-of-bias assessments

**Figure 2:**
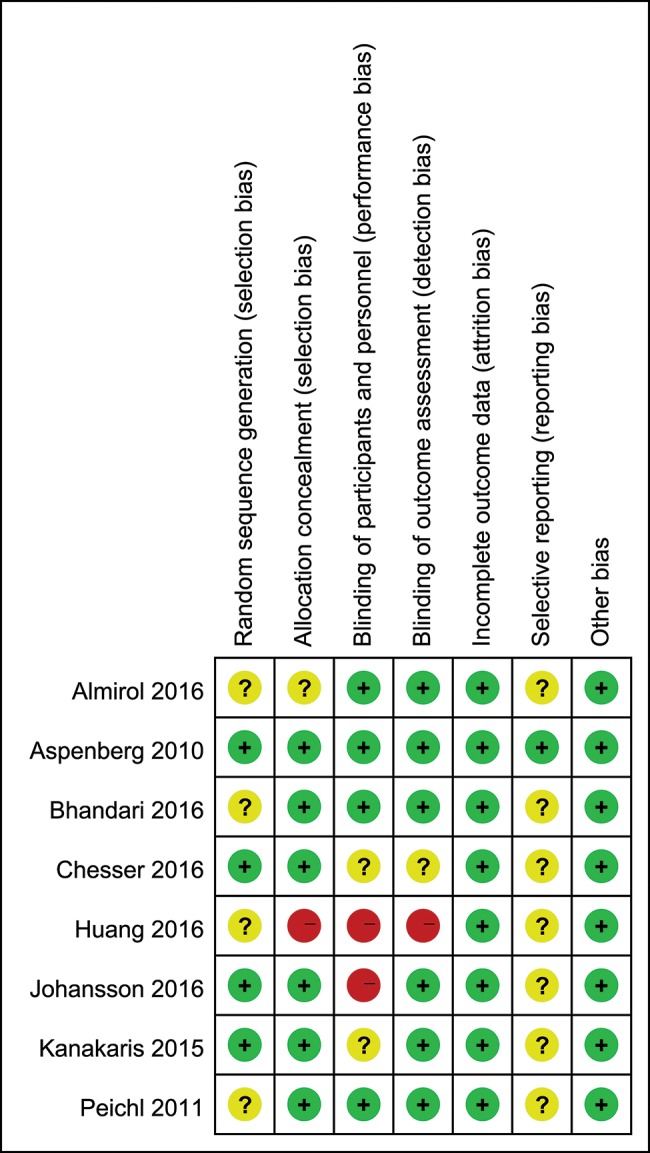
Risk-of-bias summary.

Methodological quality is shown in Figure 2. The incidence of the follow-up loss in patients was appraised, but only three trials had reported follow-up rates, which were all lower than 20%.

### Radiographic assessment of fracture healing

**Figure 3:**
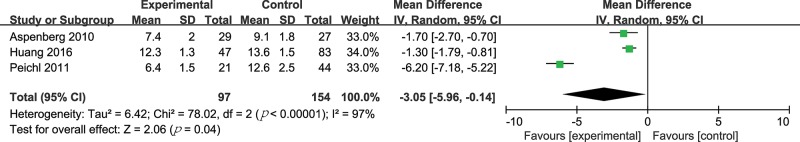
Forest plot for radiological fracture healing time.

Three trials (251 patients) compared PTH treatment with either a placebo or no treatment for the time until radiological fracture healing 31,33,34. The MD method was adopted. As shown in Figure 3, there was a statistically significant difference in fracture healing time (MD -3.05, 95% CI -5.96 to -0.14, *p*=0.04; I^2^ of heterogeneity 97%, *p*-value of heterogeneity <0.00001). A sensitivity analysis showed that the heterogeneity was significantly lower after the trial by Peichl et al. 31 was excluded (Table 3).

**Figure 4:**
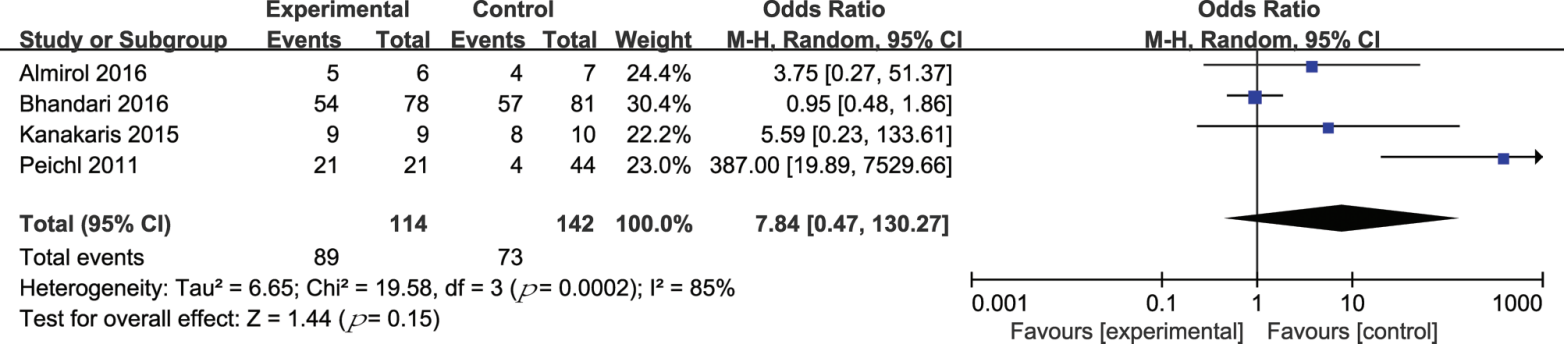
Forest plot for the radiological fracture healing rate.

Four trials (256 patients) compared PTH treatment with either a placebo or no treatment regarding the rate of radiological fracture healing 29-32. The OR method was adopted. The results are shown in Figure 4. There was no statistically significant difference in the fracture healing rate (OR 7.84, 95% CI 0.47 to 130.27, *p*=0.15; I^2^ of heterogeneity 85%, *p*-value of heterogeneity =0.0002). In a sensitivity analysis, the heterogeneity was significantly lower after the trial by Peichl et al. 31 was excluded (Table 4).

### Fracture pain degree

**Figure 5:**
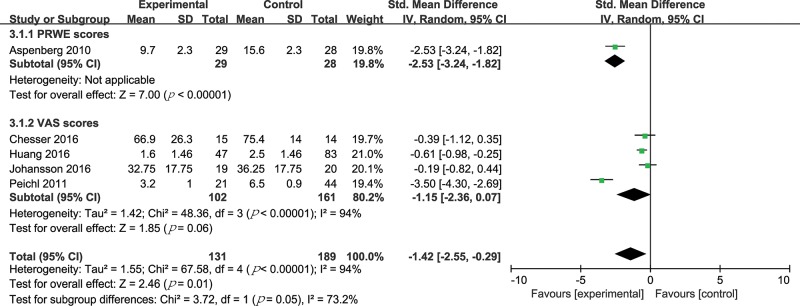
Forest plot for degree of fracture pain.

Five trials (320 patients) compared PTH treatment with either a placebo or no treatment on the degree of fracture pain by VAS or PRWE scores 31,33,34,36,37. Pain and numbness were evaluated by VAS scores in patients with trauma 31,34,36,37, while pain and function were evaluated by PRWE scores in patients with distal radius fractures 33. Due to the different scoring criteria, the SMD method was adopted. The results are shown in Figure 5. There was a statistically significant difference in fracture pain degree (SMD -1.42, 95% CI -2.55 to -0.29, *p*=0.01; I^2^ of heterogeneity 94%, *P* value of heterogeneity <0.00001). Considering the significant heterogeneity, we did a subgroup analysis on the basis of the distinct scoring methods. In the VAS score subgroup, there was still significant heterogeneity (I^2^ of heterogeneity 94%, *p*-value of heterogeneity <0.00001). We further performed a sensitivity analysis in the VAS score subgroup and found that the heterogeneity was significantly lower after the trial by Peichl et al. 31 was excluded (Table 5).

### Functional outcome

**Figure 6:**
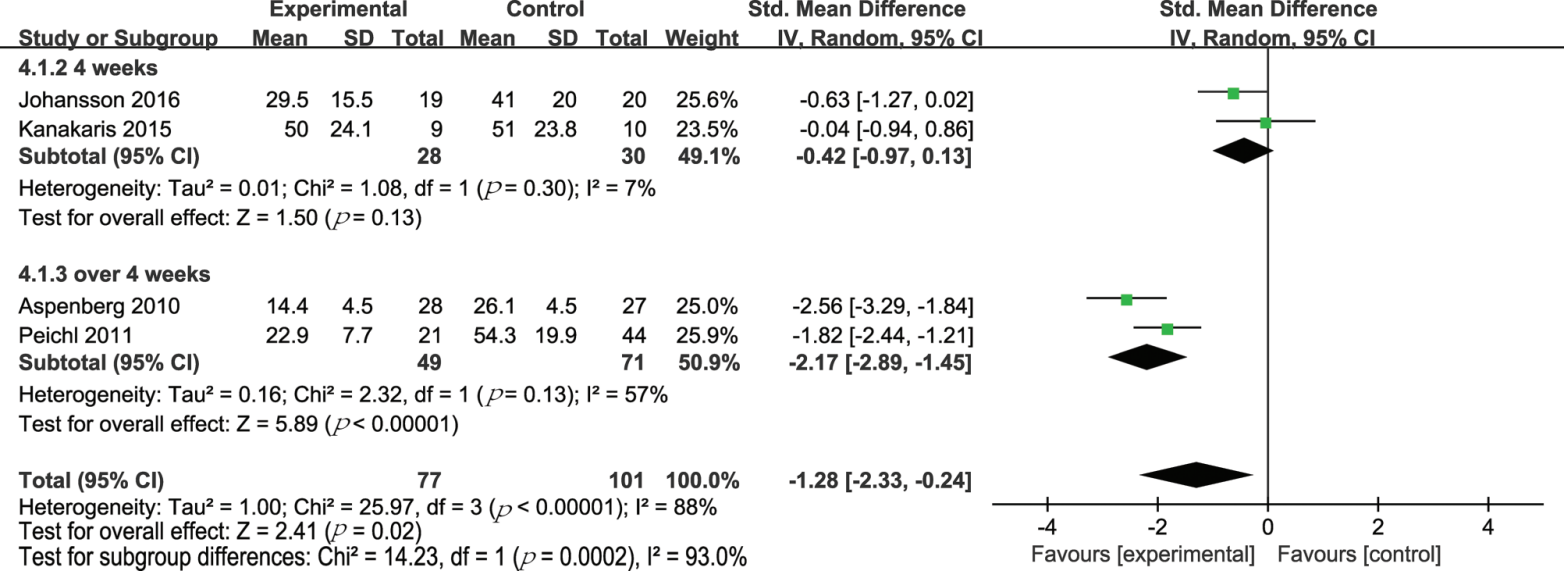
Forest plot for fracture functional outcome.

Four trials (178 patients) compared PTH treatment with either a placebo or no treatment on functional outcomes 30,31,33,37. The functional outcome was assessed with the TUG test or the self-administered PRWE questionnaire, DASH score or JHRQ 30,31,33,37. Due to the different methods, the SMD method was appropriate. The results are shown in Figure 6. The patients with PTH treatment were significantly superior to those with a placebo or no treatment in functional outcome (SMD -1.28, 95% CI -2.33 to -0.24, *p*=0.02; I^2^ of heterogeneity 88%, *p*-value of heterogeneity <0.00001). In view of the result that there was significant heterogeneity (I^2^=88%), we performed a subgroup analysis based on the duration of treatment. One group had the treatment time being equal to 4 weeks (SMD -0.42, 95% CI -0.97 to 0.13, *p*=0.13; I^2^ of heterogeneity 7%, *p* of heterogeneity =0.30) and the other group included the treatment time exceeding 4 weeks (SMD -2.17, 95% CI -2.89 to -1.45, *p*<0.00001; I^2^ of heterogeneity 57%, *p* of heterogeneity =0.13). The sensitivity analysis for the functional outcomes showed that the heterogeneity was not significantly lower after any study was excluded (Table 6). Importantly, in this meta-analysis, the functional outcomes were better when the treatment times were longer than 4 weeks.

### Adverse events

The trial by Peichl et al. declared that no deaths or adverse events were recorded 31. The trial by Huang et al. and Kanakaris et al. did not mention the relevant statistics about adverse events 30,34. Five trials reported adverse events with inconsistent methods 29,32,33,36,37. Therefore, we could not pool the relevant data, but describe them in Table 7. In comparing the PTH treatment group with a control group, there was no significant difference in light-headedness, hypercalcemia, nausea, sweating, and headache, except for slight bruising at the injection site.

### Publication Bias and GRADE Profile Evidence

**Figure 7:**
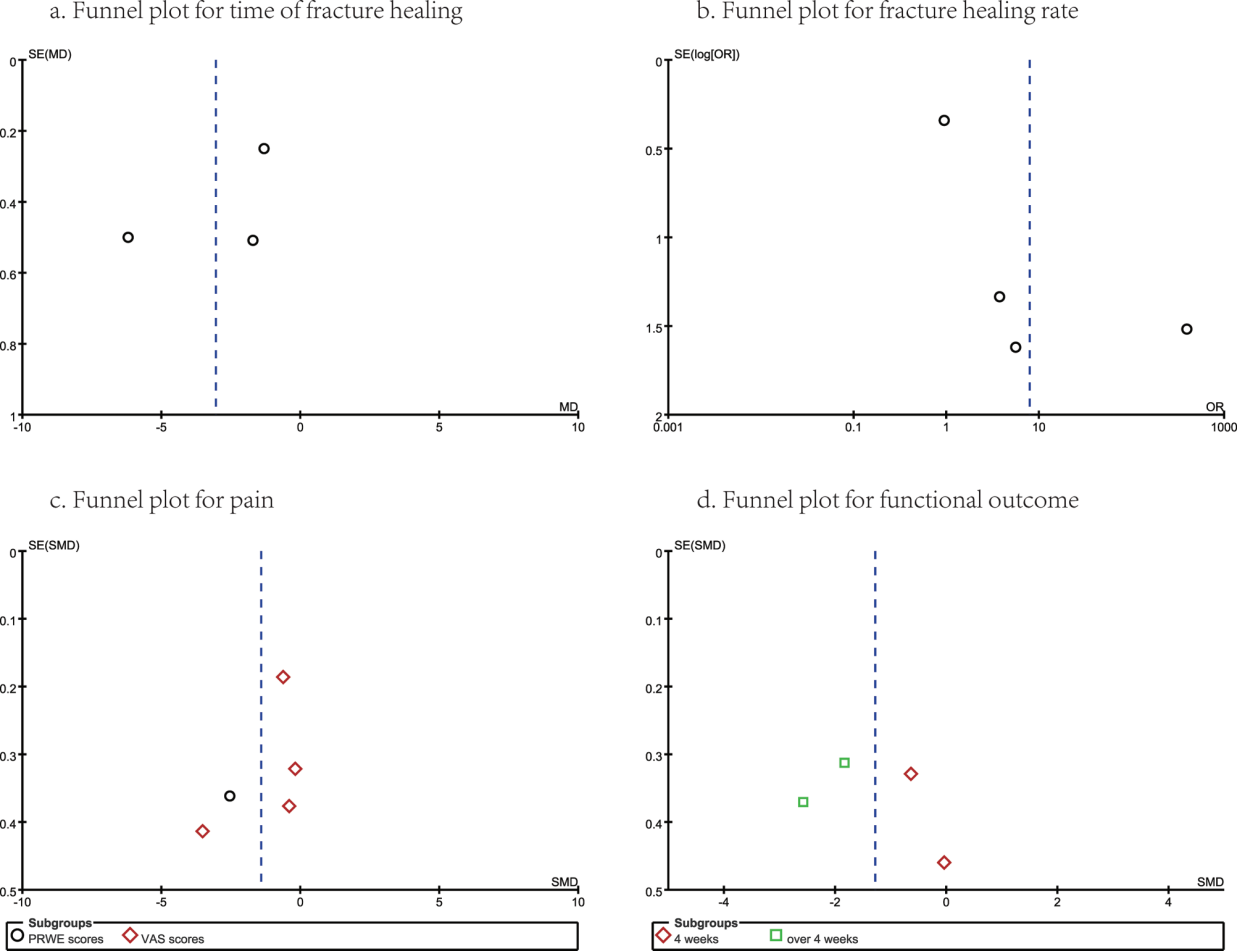
Publication bias summary.

For this meta-analysis, there was no evidence showing obvious publication bias by examining the symmetry of the funnel plots (Figure 7), but a funnel shape reference line could not be provided by the software due to the small number of studies. GRADE evidence profiles are shown in Table 8. There were no blind methods in the trials by Huang et al. and Johansson et al., no random processing in the trial by Huang et al., and inconsistent results in the trial by Bhandari et al.; these studies contrasted with the other three trials. Clearly, the most common reasons for the decreased level of evidence were the possible risk of bias and inconsistency.

## DISCUSSION

**Figure 8:**
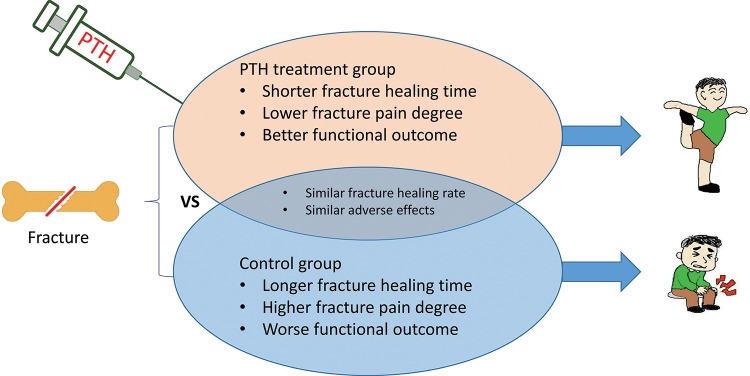
Comparison of the effectiveness and safety of parathyroid hormone in fracture healing.

In this meta-analysis, we summarized that PTH treatment in patients with fracture was better than a placebo or no treatment based on the time for fracture healing, the degree of fracture pain, and the functional outcomes (Figure 8). There is great clinical value in healing fractures over a shorter time, with reductions in pain and with functional improvements 42. Therefore, these results showed the effectiveness of PTH in fracture healing. However, previous studies were inconsistent with the effect of PTH on fracture healing 25,26. It was shown that PTH is effective in accelerating fracture healing in the study by Lou et al. 25, but not in the study by Shi et al. 26. Their studies might have some limitations, such as including the patients who received other drugs or different doses 25,26, only including the patients with osteoporosis 25, or only including fracture healing time and functional change 25. Our study included a total of 524 patients with fractures from 8 RCTs involving recently published primary studies. We included more patients and relevant outcomes, and tried to exclude inappropriate patients.

However, heterogeneity was obvious in this meta-analysis, so sensitivity analyses and subgroup analyses were performed. We found that the heterogeneity was significantly lower by exclusion of the trial by Peichl et al. 31. Fractures at different sites can be healed by different mechanisms 43. One possible explanation of the heterogeneity was that limb fracture healing differed from pelvic fracture healing in response to the anabolic effect of PTH, and we need to study these two types of fractures separately. Another possible explanation is that the equivalence of teriparatide and PTH (1-84) for fracture healing is questionable. Similarly, comparing 4-week treatment 30,37 with over 4-week treatment 31,33 produced statistically significant differences in terms of functional improvement following fracture. Although PTH can improve early callus formation 44, better effects may be observed with a longer duration of treatment. Therefore, a uniform duration of treatment is needed to support clinical decisions.

According to the results from the pooled data, PTH treatment accelerated fracture healing, which allows patients to return to normal life sooner and reduces the medical consumption and chronic morbidity associated with long-term treatment. Furthermore, PTH can be applied to any type of fracture, commenced at any time, and applied throughout the entire healing period. As a result, we suspect that PTH may be useful in the course of implant fixation and in the established nonunion fracture. Some studies have started to explore related issues 45-50, but the number of these studies is still limited, and most of them are small sample size. The hypotheses discussed here still need to be addressed by high-quality RCTs.

It was advantageous that our meta-analysis conformed to the recommendations of the Cochrane Collaboration 27 and PRISMA guidelines 28, and the quality of evidence for its outcomes was evaluated by GRADE system 40. However, there were still several limitations. First, there were only eight studies included, and the sample size was relatively small. Second, in our study, more than 79% of the fractures occurred in women, and the average age of participants was 73 years; therefore, we do not know whether the results are applicable to men or young adults. Third, it was difficult to guarantee consistent blindness because some RCTs lacked a placebo or were unclear about the “random sequence generation” and “allocation concealment”.

In conclusion, we determined that the effectiveness and safety of PTH in fracture healing is reasonably well established and credible. At this time, there are not enough studies in this field, hence we must cautiously interpret these results, and more high-quality RCTs are needed to verify the differential effects of PTH on fracture healing in different populations.

## AUTHOR CONTRIBUTIONS

Hong H and Deng Z designed the study. Deng Z conducted the study. Li J, Jiang Q and Song Q were responsible for the data collection. Hong H and Song T were responsible for data analysis. Hong H was responsible for the integration of the data analysis and manuscript drafting. Hong H and Liu Y were responsible for the data interpretation. Hong H, Liu Y, Li J, Jiang Q, Song T and Deng Z were responsible for revising the manuscript content. Hong H, Liu Y, Li J, Jiang Q, Song T and Deng Z approved the final version of manuscript.

## Figures and Tables

**Table 1:** Characteristics of the included studies.

Study ID	No. of patients	Age/years		Sex		Type of fracture
		Mean	SD	Male	Female	
Almirol et al., 2016 (32)	14	31.4	4.4	0	14	Lower-extremity stress fracture
Aspenberg et al., 2010 (33)	102	61.4	8.6	0	102	Distal radius fracture
Bhandari et al., 2016 (29)	159	70	10.5	42	117	Femoral neck fracture
Chesser et al., 2016 (36)	29	79.6	8.94	19	10	Trochanteric fractured neck of femur
Huang et al., 2016 (34)	189	81.2	8.5	61	128	Osteoporotic intertrochanteric fractures
Johansson et al., 2016 (37)	40	68	8.6	0	40	Proximal humeral fracture
Kanakaris et al., 2015 (30)	30	75	8.89	6	24	Hip fractures (low energy)
Peichl et al., 2011 (31)	65	82.8	4.1	0	65	Pelvic fracture

SD, standard deviation.

**Table 2:** Details of the interventions.

Study ID	Intervention				No. of patients in every group	Treatment time	Time of initiation
	Experimental group (eligible)	Experimental group (excluded)	Control group	Bisphosphonates group (excluded)			
Almirol et al., 2016 (32)	Teriparatide 20 μg/day calcium 1000 mg/day vitamin D 400 IU/day	-	Placebo calcium 1000 mg/day vitamin D 400 IU/day	-	6/8	8 weeks	<4 weeks
Aspenberg et al., 2010 (33)	Teriparatide 20 μg/day	Teriparatide 40 μg/day	Placebo	-	34/34/34	8 weeks	<10 days
Bhandari et al., 2016 (29)	Teriparatide 20 μg/day calcium ≤ 1000 mg/day vitamin D ≤4000 IU/day	-	Placebo calcium ≤ 1000 mg/day vitamin D ≤4000 IU/day	-	78/81	6 months	<14 day
Chesser et al., 2016 (36)	Teriparatide 20 μg/day calcium vitamin D	-	No placebo calcium vitamin D	-	15/14	42 days	10 days
Huang et al., 2016 (34)	Teriparatide 20 μg/day calcium 600 mg/day vitamin D 800 IU/day	Teriparatide 20 μg/day calcium 600 mg/day vitamin D 800 IU/day Alendronate	No placebo calcium 600 mg/day vitamin D 800 IU/day	-	47/59/83	6 months	after surgery
Johansson et al., 2016 (37)	Teriparatide 20 μg/day	-	No therapy	-	20/20	4 weeks	<10 days
Kanakaris et al., 2015 (30)	Teriparatide 20 μg/day calcium vitamin D	-	No placebo calcium vitamin D	Alendronate 70 mg calcium vitamin D	9/10/11	4 weeks	-
Peichl et al., 2011 (31)	PTH (1-84) 100 μg/day calcium 1000 mg vitamin D 800 IU	-	No placebo calcium 1000 mg vitamin D 800 IU	-	21/44	24 months	<2 days

**Table 3:** Sensitivity analyses based on various exclusion criteria for fracture healing time.

Excluded trial	No. of trials	No. of patients	Experimental group	Control group	MD (95% CI)	*p*-value for MD	I^2^,%	*p-*value for heterogeneity
Aspenberg et al., 2010 (33)	2 (31,34)	195	68	127	-3.73 [-8.53, 1.07]	0.13	99	<0.00001
Huang et al., 2016 (34)	2 (31,33)	121	50	71	-3.95 [-8.36, 0.46]	0.08	97	<0.00001
Peichl et al., 2011 (31)	2 (33,34)	186	76	110	-1.38 [-1.82, -0.94]	<0.00001	0	0.48

MD, mean difference.

**Table 4:** Sensitivity analyses based on various exclusion criteria for fracture healing rate.

Excluded trial	No. of trials	No. of patients	Experimental group	Control group	OR (95% CI)	*p*-value for OR	I^2^, %	*p*-value for heterogeneity
Almirol et al., 2016 (32)	3 (29-31)	243	108	135	10.94 [0.19, 639.47]	0.25	90	<0.0001
Bhandari et al., 2016 (29)	3 (30-32)	97	36	61	19.60 [1.00, 385.14]	0.05	68	0.04
Kanakaris et al., 2015 (30)	3 (29,31,32)	237	105	132	9.21 [0.23, 366.63]	0.24	89	<0.0001
Peichl et al., 2011 (31)	3 (29,30,32)	191	93	98	1.14 [0.56, 2.31]	0.72	2	0.36

OR, odds ratio.

**Table 5:** Sensitivity analyses based on various exclusion criteria for the subgroup of fracture pain degree by VAS scores.

Excluded trial	No. of trials	No. of patients	Experimental group	Control group	SMD (95% CI)	*p*-value for SMD	I^2^,%	*p*-value for heterogeneity
Chesser et al., 2016 (36)	3 (31,34,37)	234	87	147	-1.40 [-3.03, 0.22]	0.09	96	<0.00001
Huang et al., 2016 (34)	3 (31,36,37)	133	55	78	-1.35 [-3.33, 0.63]	0.18	96	<0.00001
Johansson et al., 2016 (37)	3 (31,34,36)	224	83	141	-1.48 [-3.16, 0.20]	0.09	95	<0.00001
Peichl et al., 2011 (31)	3 (34,36,37)	198	81	117	-0.49 [-0.78, -0.20]	0.001	0	0.51

SMD, standardized mean difference.

**Table 6:** Sensitivity analyses based on various exclusion criteria for functional outcomes.

Excluded trial	No. of trials	No. of patients	Experimental group	Control group	SMD (95% CI)	*p*-value for SMD	I^2^,%	*p*-value for heterogeneity
Johansson et al., 2016 (37)	3 (30,31,33)	139	58	81	-1.51 [-2.81, -0.20]	0.02	89	<0.0001
Kanakaris et al., 2015 (30)	3 (31,33,37)	159	68	91	-1.66 [-2.74, -0.59]	0.002	88	0.0003
Aspenberg et al., 2010 (33)	3 (30,31,37)	123	49	74	-0.87 [-1.89, 0.16]	0.10	84	0.002
Peichl et al., 2011 (31)	3 (30,33,37)	113	56	57	-1.09 [-2.55, 0.38]	0.15	91	<0.0001

SMD, standardized mean difference.

**Table 7:** Adverse effects.

Study ID	Adverse event description	No. of events in experimental group (%)	No. of events in control group (%)	*p*-value
Almirol et al., 2016 (32)	Slight bruising at the injection site	6 (100%)	0 (0%)	0.010
	Pea-sized bump below the site of fracture	1 (16.7%)	0 (0%)	0.410
	Light-headedness	0 (0%)	1 (14.3%)	0.520
Aspenberg et al., 2010 (33)	Serious adverse events	0 (0%)	3 (8.8%)	0.046
	Hypercalcemia	0 (0%)	1 (2.9%)	0.490
	Nausea	3 (8.8%)	0 (0%)	0.279
	A new distal radius fracture	0 (0%)	1 (2.9%)	0.490
Bhandari et al., 2016 (29)	Patients with > 1 adverse events	35 (45%)	40 (49%)	0.634
	Patients with > 1 adverse events possibly related to study drug	5 (6%)	5 (6%)	1.000
	Patients with > 1 serious adverse events	3 (4%)	7 (9%)	0.329
Chesser et al., 2016 (36)	None of the serious adverse effects were related to the study intervention	8 (53%)	7 (50%)	0.860
Huang et al., 2016 (34)	Not mentioned	-	-	-
Johansson et al., 2016 (37)	Nausea	3 (15.8%)	0(0%)	0.160
	Episodes of sweating	2 (10.5%)	0(0%)	0.260
	Slight headache	1 (5.3%)	0(0%)	0.470
Kanakaris et al., 2015 (30)	Not mentioned	-	-	-
Peichl et al., 2011 (31)	No adverse events or deaths were recorded	-	-	-

**Table 8:** GRADE evidence profiles for the outcomes

Outcomes	No. of Participants (studies) Follow-up	Quality of the evidence (GRADE)	Relative effect (95% CI)	Anticipated absolute effects	
				Risk with Control	Risk difference with Radiological fracture healing time (95% CI)
Radiological fracture healing time	251 (3 studies) (31,33,34)	⊕⊕⊕⊝ MODERATE due to risk of bias			The mean radiological fracture healing time in the intervention groups was 3.06 lower (6.12 lower to 0.01 higher)
Radiological fracture healing rate	256 (4 studies) (29-32)	⊕⊕⊕⊝ MODERATE due to inconsistency	OR 7.84 (0.47 to 130.27)	514 per 1000	378 more per 1000 (from 182 fewer to 479 more)
Fracture pain degree	320 (5 studies) (31,33,34,36,37)	⊕⊕⊕⊝ MODERATE due to risk of bias			The mean fracture pain degree in the intervention groups was 1.42 standard deviations lower (2.55 to 0.29 lower)
Fracture pain degree, PRWE scores	57 (1 study) (33)	⊕⊕⊕⊕ HIGH			The mean fracture pain degree, PRWE scores in the intervention groups, was 2.53 standard deviations lower (3.24 to 1.82 lower)
Fracture pain degree, VAS scores	263 (4 studies) (31,34,36,37)	⊕⊕⊕⊝ MODERATE due to risk of bias			The mean fracture pain degree, VAS scores in the intervention groups, was 1.15 standard deviations lower (2.36 lower to 0.07 higher)
Functional outcome	178 (4 studies) (30,31,33,37)	⊕⊕⊕⊝ MODERATE due to risk of bias			The mean functional outcome in the intervention groups was 1.28 standard deviations lower (2.33 to 0.24 lower)
Functional outcome, 4 weeks	58 (2 studies) (30,37)	⊕⊕⊕⊝ MODERATE due to risk of bias			The mean functional outcome, 4 weeks in the intervention groups, was 0.42 standard deviations lower (0.97 lower to 0.13 higher)
Functional outcome, over 4 weeks	120 (2 studies) (31,33)	⊕⊕⊕⊕ HIGH			The mean functional outcome, over 4 weeks in the intervention groups, was 2.17 standard deviations lower (2.89 to 1.45 lower)

The basis for the assumed risk (e.g., the median control group risk across studies) is provided in footnotes. The corresponding risk (and its 95% CI) is based on the assumed risk in the comparison group and the relative effect of the intervention (and its 95% CI). CI: confidence interval; OR: odds ratio; GRADE: Working Group grades of evidence; High quality: Further research is very unlikely to change our confidence in the estimate of the effect. Moderate quality: Further research is likely to have an important impact on our confidence in the estimate of the effect and may change the estimate. Low quality: Further research is very likely to have an important impact on our confidence in the estimate of the effect and is likely to change the estimate. Very low quality: We are very uncertain about the estimate.
